# An optimized staining technique for the detection of Gram positive and Gram negative bacteria within tissue

**DOI:** 10.1186/s13104-016-1902-0

**Published:** 2016-04-12

**Authors:** Sandra C. Becerra, Daniel C. Roy, Carlos J. Sanchez, Robert J. Christy, David M. Burmeister

**Affiliations:** Extremity Trauma and Regenerative Medicine Task Area, United States Army Institute of Surgical Research, 3650 Chambers Pass, JBSA Fort Sam Houston, TX 78234 USA

**Keywords:** *Staphylococcus aureus*, *Pseudomonas aeruginosa*, Histology, Burn, Infection, Gram’s stain

## Abstract

**Background:**

Bacterial infections are a common clinical problem in both acute and chronic wounds. With growing concerns over antibiotic resistance, treatment of bacterial infections should only occur after positive diagnosis. Currently, diagnosis is delayed due to lengthy culturing methods which may also fail to identify the presence of bacteria. While newer costly bacterial identification methods are being explored, a simple and inexpensive diagnostic tool would aid in immediate and accurate treatments for bacterial infections. Histologically, hematoxylin and eosin (H&E) and Gram stains have been employed, but are far from optimal when analyzing tissue samples due to non-specific staining. The goal of the current study was to develop a modification of the Gram stain that enhances the contrast between bacteria and host tissue.

**Findings:**

A modified Gram stain was developed and tested as an alternative to Gram stain that improves the contrast between Gram positive bacteria, Gram negative bacteria and host tissue. Initially, clinically relevant strains of *Pseudomonas aeruginosa* and *Staphylococcus aureus* were visualized in vitro and in biopsies of infected, porcine burns using routine Gram stain, and immunohistochemistry techniques involving bacterial strain-specific fluorescent antibodies as validation tools. H&E and Gram stain of serial biopsy sections were then compared to a modification of the Gram stain incorporating a counterstain that highlights collagen found in tissue. The modified Gram stain clearly identified both Gram positive and Gram negative bacteria, and when compared to H&E or Gram stain alone provided excellent contrast between bacteria and non-viable burn eschar. Moreover, when applied to surgical biopsies from patients that underwent burn debridement this technique was able to clearly detect bacterial morphology within host tissue.

**Conclusions:**

We describe a modification of the Gram stain that provides improved contrast of Gram positive and Gram negative microorganisms within host tissue. The samples used in this study demonstrate that this staining technique has laboratory and clinical applicability. This modification only adds minutes to traditional Gram stain with reusable reagents, and results in a cost- and time-efficient technique for identifying bacteria in any clinical biopsy containing connective tissue.

## Findings

### Background

In addition to metabolic functions of skin, the immune and protective capabilities of skin aid in separating the body’s external and internal environments [1]. Cutaneous tissue is the first physical barrier preventing microbial invasion of underlying host tissues. When this barrier becomes compromised (e.g., after burn injury), bacteria have the opportunity to colonize tissues, subsequently leading to the development of infection. A large variety of different types of both acute (e.g., bites, scrapes) and chronic wounds (e.g., burns, diabetic foot ulcers) are susceptible to bacterial infection [2, 3]. Approximately 7–10 % of all hospitalized patients are affected by skin and soft tissue infections [4]. Bacterial colonization of wounds inhibits and prolongs wound repair and complicates the clinical management of the patient [5]. Microorganisms can exist within wounds as either individual bacteria or as surface attached communities known as biofilms encased by a polymeric matrix composed of proteins, DNA and polysaccharides [6]. The state in which these microbes exist can have direct effects on diagnostic and treatment strategies.

*Staphylococcus aureus* and *Pseudomonas aeruginosa*, Gram positive and Gram negative microorganisms respectively, are two opportunistic pathogens that are among the most commonly associated with wounded tissue in patients [7, 8]. Reports indicate that *S. aureus* and *P. aeruginosa* play a critical role in colonizing up to 93.5 and 52.2 %, respectively, of patients with chronic leg ulcers and burn infections [9]. Burn wounds are particularly susceptible to bacterial infections due to ease of access of nutrients for colonization [10]. Specifically, burns result in both loss of the epidermal layer and the formation of dead tissue known as an eschar. The denatured proteins and lipids in the burn eschar provides an advantageous environment for bacterial growth, and the resulting infection can spread systemically and impair the wound healing process [11, 12].

Rapid diagnosis of the presence of bacteria is essential for successful treatment of infected wounds, and clinical signs of infection have proven insufficient [13]. Identification of microbes through traditional culture-based techniques is limited and time consuming. Moreover, estimations are that only ~10 % of microorganisms can be successfully cultured in laboratory conditions, suggesting that culture alone is not sufficiently sensitive [14]. Therefore, a method to identify bacteria within infected wounds that is both reproducible and has a fast turnaround time, would aid in prompt and appropriate treatment. Given the limitations of traditional culturing, advanced techniques utilizing histological and molecular-based methods have proven useful [15–17]. While molecular methods such as in situ hybridization, RT-PCR (i.e., 16S rRNA sequencing) or FACS have proven more sensitive, they are associated with high costs and long acquisition times, as well as the potential for false positives (e.g., identifying non-viable bacteria) [18–20]. Histologically, specific antibodies (i.e., immunohistochemistry) allow for the detection of individual species of bacteria, including *S. aureus* and *P. aeruginosa*. While bacterial species can be identified in this way, these stains are also time consuming and expensive. Additionally, infections are often polymicrobial, and immunohistochemistry would fail to identify multiple organisms [7, 21–23].

Recent reports have advocated for the use of the hematoxylin and eosin (H&E) stain over the Gram stain for detection of biofilms/bacteria in tissues [24, 25]. However, while existing patterns of inflammation elucidated via H&E can lead to some insight on the infection status of a wound, individual bacteria are not easily detected with H&E staining alone [26]. Alternatively, the Gram stain is able to differentiate between Gram positive and Gram negative bacteria, and is the most used technique for classifying bacterial smears in vitro. However, within tissue sections, adaptations of this technique (e.g., Brown–Brenn staining, Goodpasture method, Brown–Hopps, Steiner and Steiner stain) are needed, which lead to preferential recognition of different organisms. These modifications take advantage of unique protein profiles within different bacteria, however proteins present in the host tissue are non-specifically stained in these Gram stain processes. Consequently, discerning bacteria becomes unfeasible, particularly in cases of burn wounds in which bacteria are present within a protein-rich eschar that retains non-specific dyes in both Gram and H&E techniques [15]. In this study, we utilized a porcine burn model in which wounds were inoculated with either *S. aureus or P. aeruginosa* due to prevalence of these organisms in the clinic. Histological sections were taken to compare traditional histological stains (i.e., H&E and Gram stain), to a new modification of the traditional Gram stain. Specifically, the modification employed a collagen counterstain during the dehydration process to contrast Gram positive or Gram negative bacterial colonies with collagen-containing tissue. Finally, we tested the applicability of this staining technique by performing it on clinical biopsies from patients that underwent burn debridement.

### Methods

#### Bacterial isolates and in vitro growth conditions

A methicillin-resistant *S. aureus* clinical burn isolate was selected from strain USA300 at the US Army Institute of Surgical Research (JBSA Fort Sam Houston, TX, USA) collected as a part of patient care, but unrelated to research. *P. aeruginosa* strain PA01 is a well characterized wound isolate widely used as a laboratory strain [27–29]. *S. aureus* and *P. aeruginosa* were grown at 37 °C on blood agar plates (Remel, Lenexa, KS), and single colonies from blood agar were then inoculated into cation-adjusted Muller Hinton broth (MHB II) (Becton–Dickinson, Franklin Lakes, NJ). Biofilms were prepared as described previously [30]. Briefly, mid-logarithmic culture grown bacteria (~10^8^ CFU/mL) were diluted 1:100 with MHB II. Subsequently, 250 µL of the diluted bacteria was added to individual wells of a 24-well plate and incubated for 48 h at 37 °C under static conditions. Following growth, biofilm bacteria were detached from the 24-well plates by combined washing with sterile PBS and mechanical disruption using a pipette. Bacterial smears were prepared by applying 50 μL of the detached biofilms directly to slides followed by heat fixation.

#### Tissue collection and processing

This study has been conducted in compliance with the Animal Welfare Act, the Implementing Animal Welfare Regulations, and the principles of the Guide for the Care and Use of Laboratory Animals. Animal protocol A14-016 was approved by the Institutional Animal Care and Use Committee for United States Army Institute of Surgical Research. Skin biopsies from day 11 post-burn were kindly provided from a previously described porcine burn infected model currently under review [31]. Additionally, human samples of debrided burns were provided in a de-identified fashion by the United States Army Institute of Surgical Research Pathology Department. The use of human samples was approved by the institutional regulatory department. Both animal and human biopsies were fixed in 10 % buffered formalin for at least 48 h, and processed overnight in increasing amounts of ethanol, followed by three washes of xylene and subsequent equilibration in paraffin. Tissues were then embedded in paraffin blocks and cut into 6 μm slices. Slides were deparaffinized, cleared in xylene, and rehydrated in preparation for staining.

#### Staining techniques

Antibody specificity for *S. aureus* and *P. aeruginosa* was tested in vitro by hydrating the biofilm or planktonic smears with Hank’s balanced salt solution (HBSS). Smears were then blocked with 1 % bovine serum albumin (BSA) in tris-buffered saline (TBS) with 0.1 % Tween 20 and 0.1 % Triton X-100 for 1 h at room temperature. Smears were then incubated with the following primary antibodies for 1 h at room temperature: *S. aureus*-FITC (1:50 dilution in HBSS, ab68950, Abcam, Cambridge, UK), or *P.aeruginosa* (1:500 dilution in HBSS, ab74980, Abcam, Cambridge, UK). Following incubation, *P. aeruginosa* antibody was washed with HBSS and probed with a secondary antibody alexa-fluor 594 goat anti-chicken (1:500 dilution, ab150176, Abcam) for 1 h at room temperature. Both immunostains were completed with three washes in HBSS and subsequently mounted using ProLong Gold with 4′,6-diamidino-2-phenylindole (DAPI; Life Technologies, Grand Island, NY). In addition to antibody specific staining, Gram stain was employed in vitro using standard protocol as described by the manufacturer (Remel, Lenexa, KS).

**Fig. 1:**
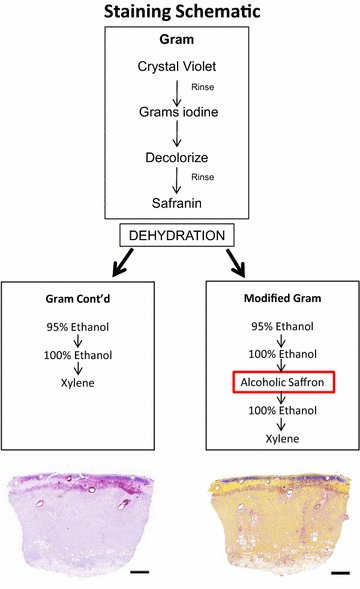
Schematic showing the modified Gram stain procedure. The traditional Gram stain procedure is completed in either case (*top box*). The major difference in the modified Gram stain procedure occurs during the dehydration process and is the application of alcoholic saffron (*lower right box*, highlighted in *red*). The *yellow* contrast in the connective tissue (collagen) within skin tissue is seen in the low magnification images on the bottom (*Scale bar* 1 mm)

For staining of normal skin and infected burn biopsies ex vivo, serial tissue sections were obtained as described above. To verify the presence of *P. aeruginosa* and *S. aureus,* immunohistochemistry was performed on these sections identically to what is described above for in vitro smears. Additionally, sections were stained with H&E (Sigma-Aldrich, St. Louis, MO) or Gram stain (Remel, Lenexa, KS). Gram stain was performed similarly to the smears described above with minor modifications (Fig. 1). Briefly, crystal violet was applied to the tissue sections for 5 min at room temperature, and slides were briefly rinsed under running tap water to remove excess crystal violet. Gram iodine mordant was applied for 2 min to the tissue sections and briefly washed in tap water. To remove any non-specific crystal violet staining, a Gram decolorizer solvent was applied to the slides for 30 s then quickly rinsed under running tap water until the water ran clear. The sections were then stained with Gram Safranin for 1 min and 40 s and followed by dehydration through a series of alcohols (95–100 %) to xylene and then coverslipped.

The modified Gram stain technique was performed in the same fashion as described in the Gram stain above, with the only addition of a counterstain after the first dehydration step of 100 % alcohol (Fig. 1). At this point, slides were immersed in alcoholic saffron (American Master Tech Scientific, Inc., Lodi, CA) for 4 min. After this incubation, the dehydration process was resumed by placing slides back into 100 % ethanol, which also allowed for removal of excess alcoholic saffron, followed by a xylene wash and coverslipping.

#### Microscopy

Images of entire wound biopsies (both porcine and human) were obtained using an AxioScan Z1 slide scanner (Carl Zeiss, Inc., Thornwood, NY) at 10× magnification. Areas of interest from serial sections were imaged using an Olympus BX60 microscope equipped with a DP71 camera (Olympus Corporation, Tokyo, Japan). Brightfield images were taken, as were fluorescent images using 594 nm (*P. aeruginosa*), 488 nm (*S. aureus*) or 405 nm (DAPI) filters. High magnification images were obtained using a 100× objective under oil-immersion.

### Results and discussion

**Fig. 2:**
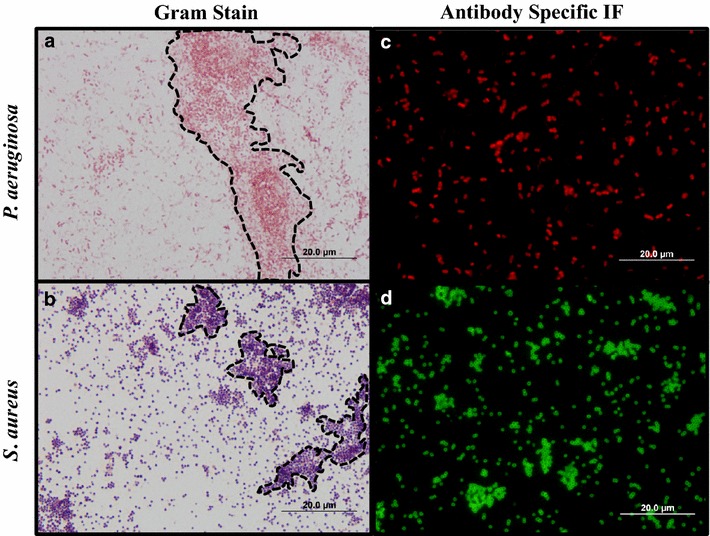
In vitro validation of applied stains. Routine Gram stain shows identification of Gram negative (**a** *P. aeruginosa)* and Gram positive (**b** *S. aureus*) bacteria in both large aggregates (*dotted lines*) and individual bacteria within the biofilm. Selected *P. aeruginosa* (**c**) and *S. aureus* (**d**)-antibodies were used to confirm species identity of cultures used for ex vivo studies

Gram stain is the most common staining technique used diagnostically within both the clinical setting and in research laboratories to differentiate between Gram positive and Gram negative microorganisms in various types of tissues [17, 26]. As seen in Fig. 2, in vitro Gram stain of *S. aureus* and *P. aeruginosa* readily differentiates between these two classes of organisms in vitro. Moreover this distinction is still evident when applied to both large aggregates and individual detached bacteria within biofilms despite the presence of structures within the polymeric matrix such as carbohydrates, lipids and proteins that have the potential to interfere with the stain [32]. For a more detailed classification of the bacterial strains used in this study, antibodies specific for *S. aureus* or *P. aeruginosa* (i.e., immunocytochemistry) were used as a validation tool. As seen in Fig. 2c, d, the shape of bacilli and cocci bacteria are illustrated, demonstrating successful identification of *P. aeruginosa* strains and *S. aureus,* respectively.

Histologically, ex vivo detection of bacterial biofilms has been completed with expensive and time consuming techniques such as electron microscopy and confocal microscopy [33, 34]. Light microscopy has been pursued for biofilm localization in conditions such as rhinosinusitis [24, 25, 35]. However, in the case of burn wounds, non-specific staining of connective tissue, cellular debris, and other proteins present within the eschar occurs due to the absorption of the crystal violet dye used in the Gram stain [15]. Also, since the eschar provides essential nutrients that support bacterial growth, it is common to find colonizing bacteria at this site [11]. In this study, we employed a porcine model of infected burns to explore a modification of the traditional Gram stain that provides contrast with connective tissue to allow for better visualization of bacteria. Pigs are the ideal choice for studying skin wounds due to similarities to human skin. For example, pig skin and human skin are similar in their epidermal and dermal thicknesses, collagen content in both papillary and reticular dermal layers, distribution of hair follicles, and healing patterns (i.e., reepithelialization as opposed to contraction) [36, 37].

**Fig. 3:**
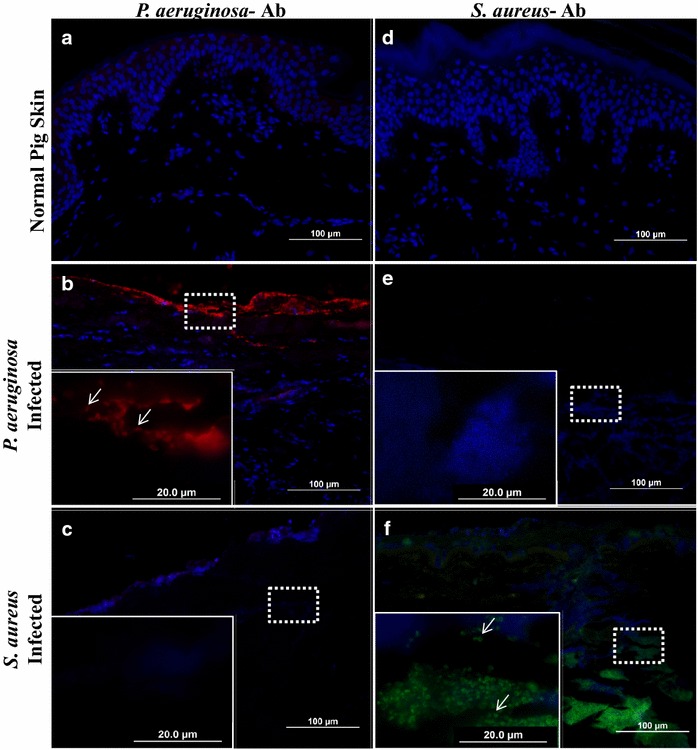
Visualization of successful bacterial inoculation ex vivo. Normal pig skin (**a**, **d**), *P. aeruginosa* infected (**b**, **e**) and *S. aureus* infected (**c**, **f**) pig skin were subjected to immunohistochemistry with either *P. aeruginosa* (**a**–**c**) or *S. aureus* (**d**–**f**) antibodies. Note that these antibodies allow for specific labeling of bacteria ex vivo as indicated by *arrows* pointing out unique bacterial morphologies

Initially, immunohistochemistry was performed with the previously verified antibodies to identify *P. aeruginosa* and *S. aureus* within burn wounds in this model (Fig. 3). Figure 3a, d show that these bacteria are not present within normal, non-burned pig skin. In Fig. 3b, we demonstrate that the *P. aeruginosa* specific antibody was able to detect *P. aeruginosa* within infected burn wounds, and are visualized as clusters of red bacilli within the superficial layers of the skin. Importantly, no reactivity of the *P. aeruginosa* antibody was seen in control pig skin, or in *S. aureus* infected samples (Fig. 3a, c). Similarly, the *S. aureus* antibody specifically labeled bacteria resent within *S. aureus* infected tissue visualized as green cocci (Fig. 3f), which was not seen in *P. aeruginosa* infected burns (Fig. 3c). Bacteria localization was also found to be different between the two species. *P. aeruginosa* localized more superficially on the burn eschar, generally an aerobic environment; whereas *S. aureus* was primarily located within/below the eschar, in a more anaerobic environment. Notably, this distribution of *S. aureus* and *P. aeruginosa* is consistent with earlier studies examining the invasiveness of these microorganisms in other models [38–41].

**Fig. 4:**
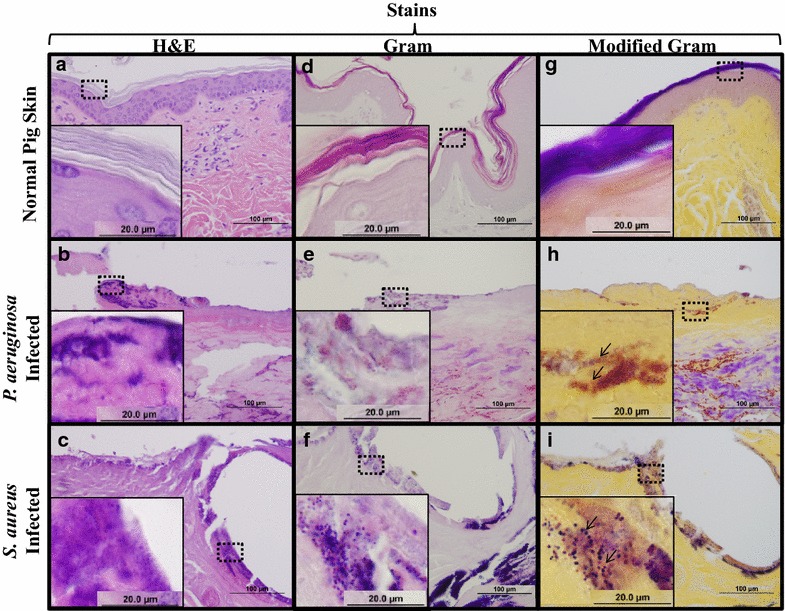
A modified Gram stain improves bacterial detection in tissue sections. Serial sections of normal porcine skin (**a**, **d**, **g**), *P. aeruginosa* infected (**b**, **e**, **h**) or *S. aure*us infected (**c**, **f**, **i**) burn wounds were stained with H&E, Gram, and Modified Gram stain. H&E of normal pig skin (**a**), *P. aeruginosa* infected (**b**) and *S. aureus* infected (**c**) pig skin illustrate coagulation of tissue, however staining within the burn eschar are not clearly identifiable as bacteria. Gram stain readily differentiates the clusters as Gram positive or Gram negative bacteria (**e**, **f**), however there is lack of contrast in the tissue in all samples. This lack of contrast is alleviated in the modified Gram, stain which distinctly enhances the detection of bacterial clusters as Gram negative (**h**) or Gram positive (**i**)

Despite the effectiveness of immunohistochemical techniques, the use of antibodies is time-consuming and expensive, and also requires prior knowledge of the bacterial species residing in the tissue of interest. Moreover, the majority of wound infections encountered are polymicrobial. Clinically, H&E is the standard staining technique used by pathologists, and recently, has been used to identify bacteria in both planktonic and biofilm forms within wounds [24, 25]. H&E staining has several attributes such as affordability and quick turnaround, as well as the ability to highlight both cells (hematoxylin) and connective tissue (eosin). However, retention of the dyes used in this stain can lead to misinterpreting clots and/or eschar as bacteria within tissue sections. As seen in Fig. 4a, H&E is effective for highlighting tissue structure and morphology in normal pig skin, with a superficial reticular dermis over the characteristic thicker fibers of the papillary dermis. After thermal injury H&E is also effective in visualizing the coagulation of collagen within the eschar (Fig. 4b, c) as individual fibers are no longer apparent. Following infection with both *P. aeruginosa* (Fig. 4b) and *S. aureus* (Fig. 4c), H&E becomes inadequate in clearly differentiating bacterial clusters from host cell debris and eschar components. This is, in part, due to the amount of dye retained within the tissue section non-specifically (i.e., the same areas of serial sections are darker after H&E sections versus the other stains). Furthermore, H&E staining is not designed to distinguish Gram positive from Gram negative microorganisms. Based on these observations, interpretation of H&E staining in tissues can be subjective and lead to inconclusive, if not incorrect, diagnoses as to the presence or absence of bacteria.

The Gram stain has also been routinely used for in vitro and clinical samples alike, where cultures from patients are collected for bacterial identification [42]. Although Gram stain is a fast, effective and inexpensive technique for bacterial differentiation, it does not allow for visualization of connective tissue (i.e., collagen) in histological samples. As seen in Fig. 4d, both cell components (e.g., nuclei) and collagen fibers are not apparent in normal pig skin. This is primarily attributed to the lack of counterstain (e.g., eosin dye in H&E staining), in which a contrasting dye used for connective tissue would be valuable for structural information. The Gram stain is able to differentiate between Gram positive and Gram negative microorganisms ex vivo, as seen in Fig. 4e, f. However, non-specific staining of damaged tissue is also apparent, which can ultimately overestimate bacterial colonization in tissues. These observations lead us to conclude that although the Gram stain is the standard staining technique in vitro, its efficiency ex vivo can lead to subjective interpretation, and also fails to illustrate important aspects of tissue morphology (i.e., collagen).

Given the limitations of the two previously described stains we sought to develop an inexpensive and fast addition to the Gram stain that highlights tissue structure. To achieve this, we modified the Gram stain by incorporating alcoholic saffron as a contrasting dye. Although a similar stain has recently been reported by Roche et al., there was no description or optimization of the technique [43]. Alcoholic saffron has been traditionally used in other staining techniques (i.e., Movat’s pentachrome) [44] for highlighting collagen fibers in tissue samples including burned skin [45]. As seen in Fig. 4g, collagen fibers within normal dermis are stained yellow with this solution, giving structural information not available following Gram stain alone. While collagen structure is greatly altered following burn injury as evidenced by coagulation seen in Fig. 4h, i, the alcoholic saffron retains the ability to dye the coagulated collagen. Importantly, this modification of the Gram stain retains the ability to distinguish both Gram positive (Fig. 4h) and Gram negative (Fig. 4i) microorganisms. The contrast provided by the alcoholic saffron allows bacteria to be detected deep within the burn eschar when compared to the Gram stain alone (Fig. 4h, e). Under low magnification, the modified Gram stain gives insight into the colonization of infected tissues by detecting differences in either red/pink retained (*P. aeruginosa*) or deep violet (*S. aureus*). At high magnification, the morphology of the bacteria (i.e., cocci and bacilli) is also easily identified.

**Fig. 5:**
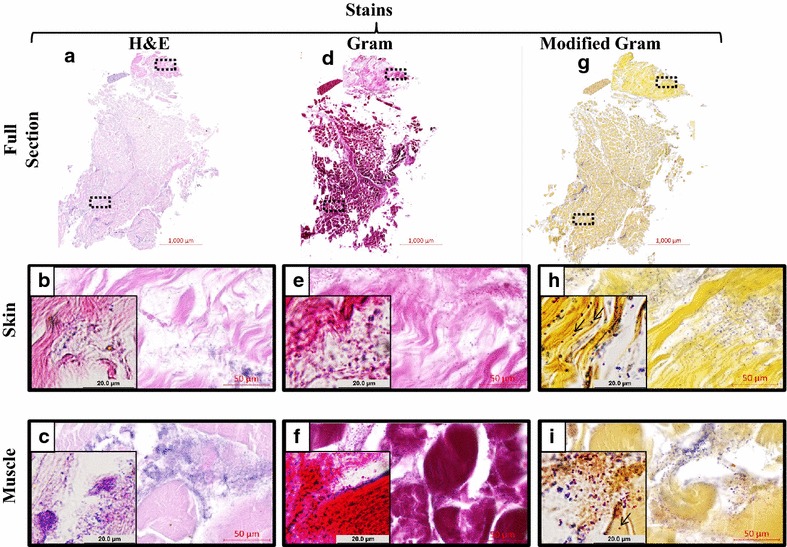
The modified gram stain applied to clinical surgical biopsies. Serial sections of tissue from a patient that underwent burn debridement were stained with three different stains. Low magnification scans of entire tissue sections show a small amount of dermis overlaying subcutaneous muscle using H&E (**a**), Gram (**b**), and Modified Gram (**c**) stains. Closer inspection of H&E stain in both skin (**b**) and muscle (**c**) areas reveal a lack of viable host cells, and hazy purple areas. Closer inspection of the Gram stain in both skin (**e**) and muscle (**f**) areas reveal some areas of bacterial morphology that are more apparent where host connective tissue is more sparse. Closer inspection of the modified Gram stain in both skin (**h**) and muscle (**i**) illustrates a counterstain that allows for identification of bacterial morphology (*arrows*) within connective tissue. Magnification *insets* are ×100

In order to test the clinical applicability of the modified Gram technique, the same staining comparison was performed on surgical biopsies from a de-identified patient (Fig. 5). The selection criteria for the biopsy were for a patient undergoing burn debridement, identified as having a polymicrobial infection. At low magnification, H&E, Gram, and modified Gram (Fig. 5a, d, g, respectively) revealed a small amount of dermis overlaying a substantial amount of subcutaneous muscle. Higher magnifications of the H&E stain showed retention of Hematoxylin (purple) in both the skin (Fig. 5b) and muscle (Fig. 5c) areas without readily identifiable bacterial morphology. Of note, dye retention during the Gram stain process led to non-specific staining, especially in the skeletal muscle (Fig. 5f). This was slightly less apparent in the dermis (Fig. 5e), which allowed for bacteria visualization in areas of less dense host tissue. However, high magnification of the modified Gram stain clearly demonstrated bacterial morphology within dermal connective tissue (Fig. 5h) and subcutaneous muscle (Fig. 5i).

While a wide variety of molecular techniques (e.g., 16S rRNA pyrosequencing, in situ hybridization, RT-PCR) are now available, it will take time before these technologies may get sufficiently refined to allow for fast turn around and become clinically feasible. However, histopathology will always have a place for diagnosis of different tissues, and a quick, inexpensive, detailed, and reproducible histological technique for bacteria identification and localization is desired. The modified Gram stain we have described herein improves on the traditional Gram stain by providing contrast of connective tissues. The incubation time in alcoholic saffron for these tissues was found to be optimal at 4 min. Less time led to lighter staining of the connective tissue, while longer incubation in alcohol began to remove the Gram stain components. This 4 min incubation represents a short addition to the staining process already in clinical use. While in this study we utilized paraffin embedding that takes several days of processing, a flash-freezing strategy could potentially allow for bacterial identification in less than an hour from biopsy collection [46].

There are potential limitations for implementing this stain. For example, certain species (e.g. fungi, acid fast bacteria, and other Gram variable bacteria) may not be highlighted using the Gram stain. However, the applications examined herein were aimed at addressing clinically relevant species of bacteria most often associated with wound infections. As such, these most common species present within chronic non-healing wounds would be detected with the technique described herein. Additionally, this staining technique does not provide any evidence for the species of bacteria present in tissue, which would need to be addressed with subsequent diagnostic tools. Also, tissues used in the current study have been optimized for infection which may make detection easier compared to a lesser amount of bacteria that may be present in an acute wound. Determining whether or not wounds that contain fewer bacteria would benefit from this stain is a point of further study.

### Conclusions

We have described a histological technique that allows for visualization of tissue structures along with detection of Gram positive and Gram negative bacteria. An important advantage to the modified Gram stain is its ability to highlight collagen, making it applicable to any tissues that contain collagen. Employing this technique in other collagen-containing tissue samples may require optimization of staining times based on the collagen content of that tissue sample. As cutaneous tissue is very rich in dense collagen fibers, staining times may be even shorter for other tissues in which collagen is less abundant and/or contained within certain locations (e.g., the lamina propria). We have demonstrated the clinical relevance of this modification to the Gram stain, in that it is capable of visualizing bacteria within burn wound samples from both the laboratory and the operating room. Moving forward this technique can be easily implemented in the clinic, allowing for rapid examination of the status of infection in tissue samples.
